# Case Report: Clostridial Gas Gangrene of Pelvic Wall After Laparoscopic Rectal Cancer Surgery Induced Fatal Sepsis

**DOI:** 10.3389/fsurg.2022.822605

**Published:** 2022-03-18

**Authors:** Xing Wang, Simin Jiao, Zhen Sun, Zhicheng Wang, Xudong Wang, Tianzhou Liu

**Affiliations:** Department of Gastrointestinal Surgery, The Second Hospital of Jilin University, Changchun, China

**Keywords:** fatal sepsis, postoperative infection, laparoscopic rectal cancer surgery, clostridial gas gangrene, subcutaneous emphysema

## Abstract

Clostridial gas gangrene is an unusual but fast-spreading necrotic infection of soft tissue relevant to high mortality rates. We report a case of postoperative gas gangrene of the pelvic wall, scrotum, and perineal site, with very acute onset and rapid progression of symptoms after laparoscopic radical resection for rectal cancer. Although potentially treatable with appropriate antibiotic cover and urgent thorough surgical debridement, this case still developed irreversibly into fulminant and fatal sepsis. The possible etiologic factors, better measures of diagnosis, and treatment are discussed, and the relevant literature is reviewed.

## Introduction

Clostridial gas gangrene (CGG) is a rare necrotizing infection of the skeletal muscles. Classically, the CGG is mainly caused by autogenous or exogenous clostridial infection (1). While more frequently associated with food poisoning and traumatic events like open surgery (2), uncommon reports of spontaneous non-traumatic gas gangrene following laparoscopic surgery have also been recorded. A laparoscope, based on its advantages of clear vision, less injury, and quick recovery, is naturally favored by surgeons and patients. Notably, it is reported that most cases of CGG after laparoscopic surgery share tight bonds with underlying immunodeficiency diseases such as malignancy, hematological disease, and diabetes mellitus (3). Moreover, it is typically manifested as a life-threatening process and requires immediate emergency care. However, an early definitive diagnosis of it arising in the abdominal or pelvic wall is often tough and time-consuming.

Here, we describe a rare case of CGG following a laparoscopic radical resection for rectal cancer in which gas was detected in the soft tissues extending from the pelvic wall, scrotum, and perineal site, and even in the upper abdomen by imaging tests and physical examination. To our knowledge, this is the first time that CGG of the pelvic wall, scrotum, and perineal site after minimally invasive rectal surgery of tumor-specific mesorectal excision (TSME) and Clostridium perfringens revealed by Next-generation sequencing (NGS) of peripheral blood sample have been described. Literature is reviewed in reference to the present diagnosis and treatment modalities, as well as the possible preventive measures relevant to laparoscopic surgery.

## Case Report

An overweight 65-year-old male patient, who suffered complaints of abdominal pain for 2 weeks, underwent laparoscopic rectal cancer surgery. His medical history revealed severe hypertension of level 3, diabetes mellitus type 2, lacunar cerebral infarction, and duodenal ulcer. The previous colonoscopy showed an ulcerative mass at the junction of the rectum and sigmoid colon; pathological analysis revealed the moderately differentiated adenocarcinoma; PET evaluated it as rectal cancer along with the invasion of serosa and multiple metastases of the peripheral lymph node.

Consequently, the patient was scheduled for laparoscopic surgery to resect this mass. It was gratified that the patient was very agreeable to our procedure plan. After routine intestinal preparation with oral laxatives, laparoscopic surgery was fully planned with preoperative intravenous use of Etimicin, given the allergic history to cephalosporins. The laparoscopic surgery procedure was performed in 2 h and 40 min under general anesthesia without complications: the TSME procedure was selected for this patient; the specimen was retrieved through the smaller lower abdominal incision of ~6 cm with wound protector; the double stapling technique (DST) was applied to anastomosis; two abdominal drainage tubes were placed in the pelvic cavity pulled from the lower bilateral trocar site, and the anus tube was set as usual.

**Figure 1:**
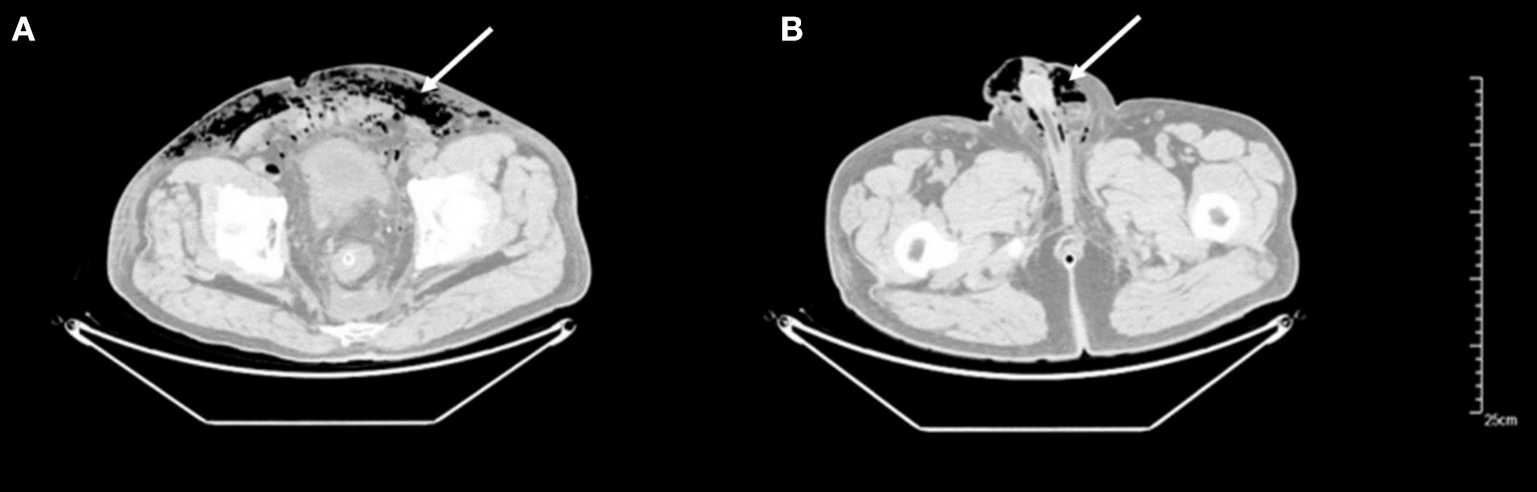
CT imaging (axial views) demonstrating subcutaneous gas of lower abdomen **(A)** and scrotum **(B)** indicated by white arrows.

The initial postoperative course was uneventful with conventional fasting, fluid infusion, and gastrointestinal decompression. However, 24 h after the surgery, the patient started complaining of incisional pain in the lower abdomen. There was no obvious abnormality during the process of dressing change. On the following night, the patient began to complain of severe low abdominal pains, palpitation, and general malaise accompanied by slight abdominal distension, which deteriorated continuously until the next morning. His vital signs revealed a heart rate of 110 beats per minute, blood pressure 122/76 mm Hg, temperature 36.9°C, respiratory rate 38 per minute, and oxygen saturation of 88% on room air. The patient appeared restless with dyspnea and tachycardia, and there was a small amount of bloody liquid in the drainage tube. Initial local symptoms included severe pain disproportional to local findings over the affected area of the scrotum and perineal region besides the incision. The physical examination was notable for discoloration and swelling of the pelvic wall near the incision involving the scrotum and perineum: initial bronzing of the skin progressed from pale erythema with induration remarkable to purple ecchymosis with a foul odor and spreading palpable crepitus. An urgent CT scan of the chest, abdomen, and pelvis suggested subcutaneous pneumatosis of the abdomen especially the pelvic wall and bilateral scrotum, with a small amount of fluid observed in the pelvic cavity (Figure 1). The rectum anastomotic leakage and intra-abdominal sepsis were excluded. At the same time, blood gas analysis implied type I respiratory failure, severe lactic acidosis, and hyperkalemia, and a score of Laboratory Risk Indicator for Necrotizing Fasciitis (LRINEC) tool (4) is compiled by using its parameter, including hematological and biochemical parameters: the white cell count, hemoglobin, sodium, glucose, serum creatinine, and C-reactive proteins, which yielded quite a high risk of necrotizing infections with the maximum score.

**Figure 2:**
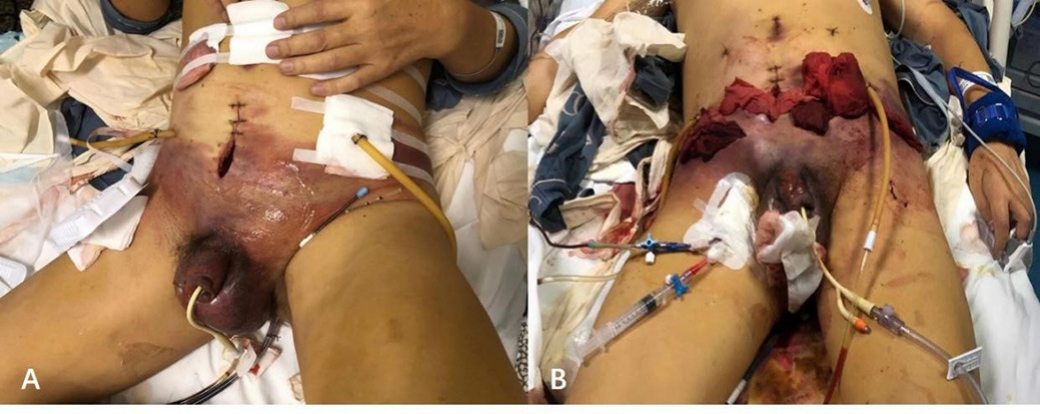
Typical skin discolorations and swelling of pelvic and scrotum with dishwasher-like fluid before **(A)** and after **(B)** debridement.

Combined, an immediate decision was taken to explore and decompress the pelvic wall and scrotum for suspected necrotizing fasciitis. Multiple deep incisions including the bilateral lower abdomen and scrotum were performed with gauze drain, and the debridement space was expanded deeply by fingers at the bedside. On the surface, there was a large amount of dishwasher-like exudation extending along the fascial planes, making the thick dressing wet as is shown in Figure 2. In addition, the patient was closely observed and commenced on high-flow oxygen, antibiotic therapy with meropenem, and intravenous fluid resuscitation.

Considering the severe condition, the patient was transferred to the intensive care unit to strengthen the monitoring and support treatment. Supposed that the necrotizing fasciitis or CCG cannot be ruled out, the isolation ward was a necessity. Adversely, in the following 10 h, septic shock ensued, for which active anti-infection treatment for a specific pathogen was taken, and blood purification treatment was applied to treat sepsis, hyperkalemia, and acidosis. However, the health status of this patient still went from bad to worse. The hemodynamic status and respiratory function declined constantly, necessitating cardiotonic and mechanical ventilation. A great number of vasopressor drugs still failed to maintain blood pressure from declining and the heart rate decreased with a segmental rhythm. Despite active treatment of CPR and intravenous adrenaline, the patient showed no signs of recovery and was declared deceased in the afternoon of POD2.

### Investigations

**Figure 3:**
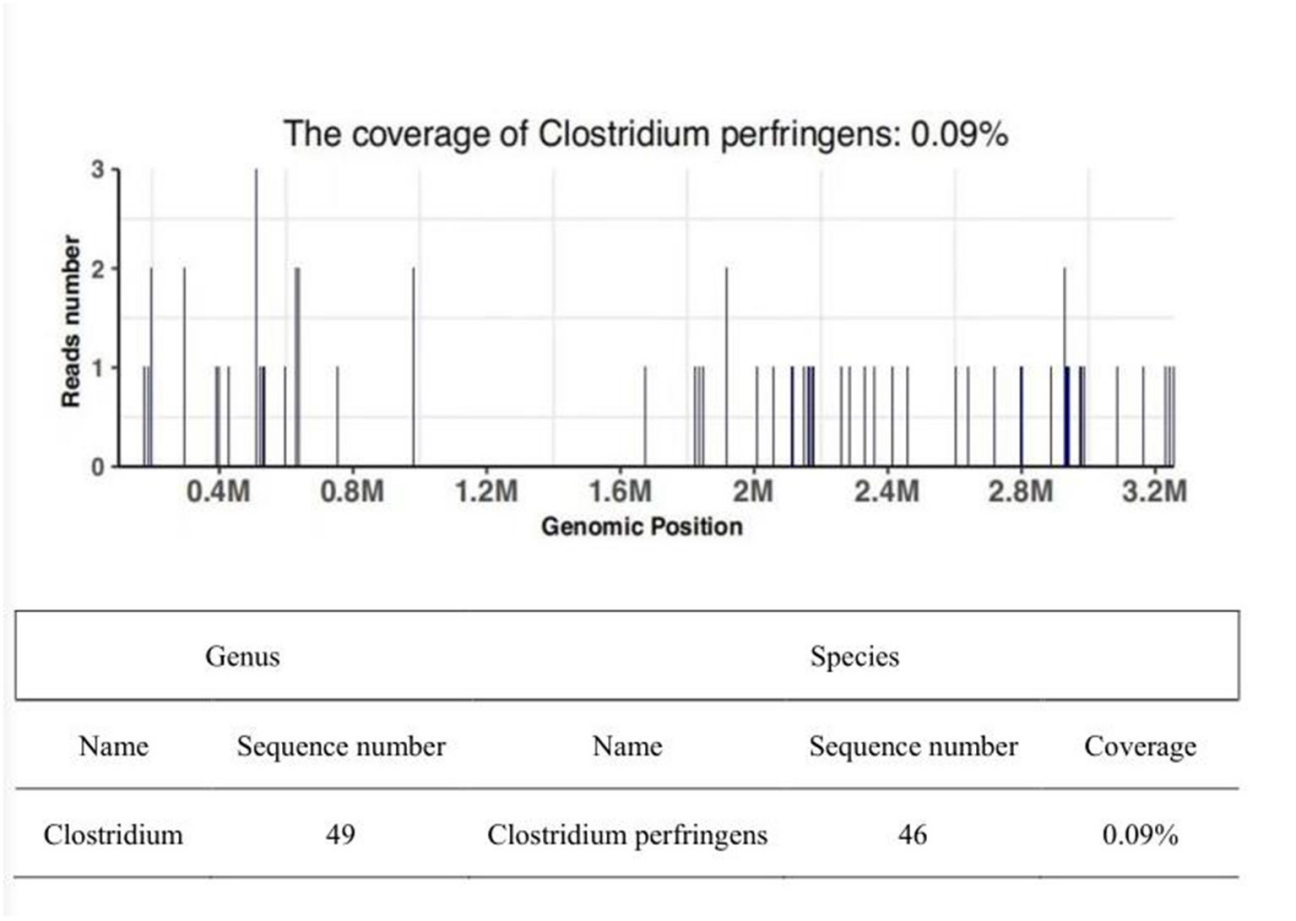
Sequencing of Clostridium perfringens yielded a total coverage of 0.09 % in the peripheral blood by NGS. The sequence number of the strict comparison of the microorganism detected at the level of genus/species.

Apart from the CT scan of the pelvis which showed multiple gas bubbles, the discharge was examined by microscope: plentiful infiltrated leucocytes and Gram-positive bacilli of a large rod were discovered. However, there was no growth of bacteria found from ordinary and enriched culture. On discussion with the microbiologist, it was concluded that the Gram-positive bacilli were most likely an anaerobe to be cultivated in the anaerobic medium. Fortunately, we also sent the peripheral blood samples to perform NGS to specify this kind of bacilli, and 2days later, we obtained an exact result that the number of unique reads, coverage, and depth of the identified pathogen sequences was 46.09%, and 1, respectively. It meant that Clostridium perfringens were in the peripheral blood sample and confirmed our diagnosis of CGG with higher sensitivity (Figure 3).

The status of the patient was not improving but even getting worse with time, and the change of parameters associated with multiple organ failure had worsened gradually during the clinical course in this patient (Table 1).

**Table 1:** Parameters reflecting severe infection during the clinical course.

		**POD2 Afternoon**	**POD2 Morning**	**POD1 Morning**	**Pre-operative Day**
Parameter	Normal range	Septic shock; CGG	
WBC	3.5–9.5 × 10^9^ /L	—	33.9	14.7	8.4
Hb	130–175 g/L	—	72	125	134
PLT	125–350 × 10^9^/μL	—	138	227.9	246.6
AST	15–40 U/L	760	780	10	14
ALT	9–50 U/L	90	120	8	9
T.Bil	2.0–20.1 μmol/L	181.32	236.30	19.95	13.45
LDH	120–250 U/L	6,890	7,580	—	—
CPK	50–310 U/L	4,580	4,870	—	—
Cre	57–111 μmol/L	190	74	95	88
hs-CRP	<5.0 mg/L	60.71	53.54	—	—
PCT	<0.5 ng/mL	13.6204	15.0284	—	—
APTT	25.4–38.4 s	41.1	—	28.2	29.7
TT	10.3–16.6s	17.3	—	13.2	13.7
FDP	0–10 μg/mL	29.2	—	9.8	4
D-dimer	0–1 μg/mL	11.45	—	3.50	0.59

*WBC, white blood cells; Hb, hemoglobin; PLT, platelet; FDP, fibrinogen degradation product; AST, aspartate aminotransferase; LDH, lactate dehydrogenase; T.Bil total bilirubin; CPK, creatinine phosphokinase; Cre, creatinine; hs-CRP, hypersensitive C-reactive protein; CGG, Clostridial Gas Gangrene*.

### Differential Diagnosis

There was uncertainty as to whether this is a common finding of subcutaneous emphysema postoperatively or, indeed, an infective complication especially without the eventful appearance of skin; but, even if there is a clear change of abdominal or perineal skin in color or texture, we still need to differentiate anastomotic fistula from infections including simple cellulitis, necrotizing fasciitis or CGG by CT, and other diagnostic measures.

Based on this abnormal postoperative presentation, we undertook a literature search to identify any further cases of Clostridium perfringens infection of the pelvic wall following laparoscopic surgery. Using the Medline, Web of Science databases, and Gray Literature Report database for unpublished work, we conducted a search with only one more reported case of CGG, but in a different involved area of mainly pelvic cavity following laparoscopic surgery of rectal carcinoma, was found (5).

## Discussion

Postoperative infections are one of the most common complications in general surgery, and rates have been reduced with the routine administration of perioperative antibiotics (6). The CGG, a necrotizing tissue infection, is reportedly more common after penetrating trauma or major surgery and is rarely reported without obvious predisposing events. Moreover, the timing of symptom onset postoperatively can vary a lot, with reports ranging from hours to days (7). Patients with necrotizing infections of any type often present with edema, fever, malaise, and pain out of proportion to exam findings—this subtle presentation increases the risk of delayed diagnosis, and high clinical suspicion is essential (8). Previous documents once reported Gas Gangrene or necrotizing fasciitis after an open rectal surgery or laparoscopic cholecystectomy (5, 9). However, they varied a lot according to different severities of operation trauma, various surgical areas, and distinct nature of the infection. Thus, this is the first time CGG of the pelvic wall, after laparoscopic radical resection for rectal cancer, and it is what this case is about.

Although the pathogenesis of CGG is still unknown to some extent, there must be a relatively hypoxic tissue where clostridial and other pathogens may accumulate and proliferate. The relative hypoxic tissue can be created as the pneumoperitoneum with positive intra-abdominal pressure and a constant flow of carbon dioxide (CO_2)_ without oxygen (O_2)_ that reduces blood flow in the abdominal and pelvic wall. The deep and small abdominal incision of just 5–6 cm with specimen retrieval bag and trocar incision shortened the exposure time and strengthen the hypoxic environment. Then, subcutaneous emphysema may form and develop in these places, creating an environment in which both aerobic and anaerobic bacteria could thrive. As is known to all, the Gram-positive, obligate anaerobe, and Clostridium perfringens are well-known pathogens that form part of both the environmental and gastrointestinal flora (10). Hence, the surgical region of the rectum adjacent to the pelvic and perineal area is subject to exogenous or endogenous infection from bacteria. As for the specific infective pathway, considering there are adequate preoperative preparation, definite anastomosis, and no fistula after resection, damaged and contaminated tissue during surgery may not be the ideal candidates. Some researchers considered the trocar port as a high-risk area where the surrounding muscles could be damaged by “capacitive coupling”: using extensive coagulation, the transferred electricity/heat through the trocar induces damage to neighboring tissues (9). When port-site infections occur, the pathogens might inoculate the other areas in different ways. For example, pelvic drainage tubes pulled from the lower bilateral trocar site may serve as an important way for pelvic infection. Furthermore, the fatal outcome may be partly related to this patient's underlying basic diseases. Known predisposing conditions involved poorly controlled diabetes, obesity, malignancy, malnutrition, intravenous drug use, and cirrhosis of the liver (3).

With the infection of subcutaneous tissues, rapid bacterial growth and clostridial toxin production occur. These toxins, most notably alpha and theta, facilitate the formation of vascular thrombi and cell lysis, thereby further augmenting tissue hypoxia, subsequent necrosis, and emphysema formation, which constitutes a vicious cycle. The synergistic effect of anaerobic and aerobic organisms may explain the potential for fulminant disease: severe sepsis in our patient caused a series of chain reactions including hemolytic anemia, jaundice, hemoglobinuria, hyperkalemia, and acidosis, combined with increased leukocytes and procalcitonin, also seen in other reports (11, 12). There were extremely limited time and a slim chance of complete and thorough debridement due to the rapid progressive process. Subsequently, septic shock and multiple organ failure can rapidly lead to death particularly in untreated patients.

Diagnosing this uncommon complication seems difficult in the early stages when symptoms mimic those of common cellulitis, abscess formation, necrotizing fasciitis, or peritonitis (11). Early findings were consistent with necrotizing fasciitis with a loss of sheen and texture of the fascia and pus extending along the fascial planes. The CCG has a lot in common with subcutaneous emphysema, a typical occasion following laparoscopic surgery, hence, it is easy for it to escape diagnosis by an inexperienced surgeon. Not until the result of CT and secretion smear come out can the diagnosis of CGG be confirmed. The enhanced level of creatine kinase, a symbol of muscle involvement, and the change of brick red-cooked muscle after incision also have certain diagnostic meanings to imply the involved level.

The gold standard of diagnosis is the frozen-section biopsy, in which a portion of skin/subcutaneous tissue is excised under local anesthesia and is examined for the presence of bacteria. Whereas, it is effective but usually difficult in sampling when crowded with too much infective necrotic tissue. Anaerobic culture of tissue or discharge may not be prepared routinely in most departments, but of great diagnostic value, especially when necrotizing fasciitis, even when CGG is suspected. With the rapid development of NGS, it plays an increasingly irreplaceable role in microbial testing with characteristics of convenience, higher accuracy, efficiency, and resolution. The NGS test of the blood sample from our patient implies some fragments of Clostridium perfringens, yielding a high-risk index of CGG.

Additionally, MRI and necrotizing skin infections detected using the tool LRINEC also facilitate THE diagnosis, but whichever diagnostic procedures will be chosen should not delay the surgical intervention. Notably, the bacterial smear, culture, and NGS are all direct and effective ways to determine the presence of bacteria. Thus, it is of great value and meaning to make an objective evaluation of NGS result with clinical manifestation, to remind the laboratory staff of anaerobic culture of tissue fluid or secretion, and if necessary, to remove the tissue for pathological examination and to make full use of LRINEC tools or otherwise.

Once the diagnosis is confirmed, rigorous excision of necrotic, and affected tissue should be performed without delay. The thorough surgical debridement cannot be placed on more emphasis; delaying surgery has been shown to considerably increase mortality in case series (13). Under circumstances when the initial debridement was radical, the patients succumbed, whereas those with successful resection of the necrotic tissue survived. This may be another essential prognostic factor for survival besides the time and affected area (14). In this case, we performed an urgent incision and tension reduction in obviously swollen areas at the bedside, while did not perform complete emergency debridement, mainly because of the patient's poor general condition and abnormal coagulation function. Compared with the top priority of basic life support like endotracheal intubation, the time-consuming and highly-demanding extensive debridement should be put aside temporarily.

Additional broad-spectrum antibiotic therapy is also necessary, as CGG is usually caused by a variety of pathogens, both aerobic and anaerobic. Historically, penicillin and metronidazole were the drugs of choice; however, clindamycin and erythromycin have greater efficacy against Clostridium (15). At this time, we chose a powerful drug, meropenem targeted at Clostridium. Other supportive treatments may include intensive care unit admittance, hemodialysis, parenteral feeding, and mechanical ventilation. There have been no randomized studies to illustrate the role of hyperbaric oxygenation therapy (16).

## Conclusion

Laparoscopic surgery has increasingly become the first choice of treatment for rectal cancer. Taking pneumoperitoneum, port-site infection, and multiple predisposing factors into account, we should focus on all cases of sudden deterioration after laparoscopic rectal surgery, maintain a high index of suspicion, and request early senior review for fear of this rare but potential implication. Moreover, the NGS and anaerobic culture should not be ignored, and a thorough surgical debridement ought to be performed without delay, otherwise, it can progress sharply. Even though at the end of fulminant and fatal sepsis, our case is still worth further investigation and discussion.

## Data Availability Statement

The original contributions presented in the study are included in the article/supplementary material, further inquiries can be directed to the corresponding author.

## Ethics Statement

Written informed consent was obtained from the individual(s) for the publication of any potentially identifiable images or data included in this article.

## Author Contributions

XiW and TL initiated the study and drafted the manuscript. TL and XuW performed the surgery with the assistance of SJ, ZS, and ZW. All authors revised and approved the manuscript.

## Conflict of Interest

The authors declare that the research was conducted in the absence of any commercial or financial relationships that could be construed as a potential conflict of interest.

## Publisher's Note

All claims expressed in this article are solely those of the authors and do not necessarily represent those of their affiliated organizations, or those of the publisher, the editors and the reviewers. Any product that may be evaluated in this article, or claim that may be made by its manufacturer, is not guaranteed or endorsed by the publisher.
